# Hemopoietic Cell Kinase amplification with Protein Tyrosine Phosphatase Receptor T depletion leads to polycythemia, aberrant marrow erythoid maturation, and splenomegaly

**DOI:** 10.1038/s41598-019-43373-6

**Published:** 2019-05-07

**Authors:** Matthew Ku, Ruth N. MacKinnon, Meaghan Wall, Nisha Narayan, Carl Walkley, Heung-Chin Cheng, Lynda J. Campbell, Louise E. Purton, Harshal Nandurkar

**Affiliations:** 1 Department of Haematology, St Vincent’s Hospital, 3065 Fitzroy, Australia; 2 Victorian Cancer Cytogenetics Services, St Vincent’s Hospital, 3065 Fitzroy, Australia; 3 St Vincent’s Institute of Medical Research, 3065 Fitzroy, Australia; 4 Department of Medicine, St Vincent’s Hospital, The University of Melbourne, 3065 Fitzroy, Australia; 5 The University of Melbourne, 3010 Parkville, Australia; 6 The Australian Centre for Blood Diseases, Monash University, 3004 Melbourne, Australia

**Keywords:** Myeloproliferative disease, Cancer genetics

## Abstract

Deletion of long arm of chromosome 20 [del(20q)] is the second most frequent recurrent chromosomal abnormality in hematological malignancies. It is detected in 10% of myeloproliferative neoplasms, 4–5% of myelodysplastic syndromes, and 1–2% of acute myeloid leukaemia. Recurrent, non-random occurrence of del(20q) indicates that it is a pathogenic driver in myeloid malignancies. Genetic mapping of patient samples has identified two regions of interest on 20q – the “Common Deleted Region” (CDR) and “Common Retained Region” (CRR), which was often amplified. We proposed that the CDR contained tumor suppressor gene(s) (TSG) and the CRR harbored oncogene(s); loss of a TSG together with over-expression of an oncogene favored development of myeloid malignancies. Protein Tyrosine Phosphatase Receptor T (PTPRT) and Hemopoietic cell kinase (HCK) were identified to be the likely candidate TSG and oncogene respectively. Retroviral transduction of HCK into PTPRT-null murine LKS+ stem and progenitor cells resulted in hyperproliferation in colony forming assays and hyperphosphorylation of intracellular STAT3. Furthermore, over half of the murine recipients of these transduced cells developed erythroid hyperplasia, polycythemia and splenomegaly at 12 months, although no leukemic phenotype was observed. The findings suggested that HCK amplification coupled with PTPRT loss in del(20q) leads to development of a myeloproliferative phenotype.

## Introduction

Deletion of the long arm of chromosome 20 [del(20q)] is the second most frequent recurrent chromosomal abnormality in hematological malignancies^1^. It is associated with myeloid malignancies, detected in 10% of myeloproliferative neoplasms (MPN)^2^, 4–5% of myelodysplastic syndromes (MDS)^3^, and 1–2% of acute myeloid leukemia (AML)^4^. Del(20q) can result from a simple deletion or an unbalanced translocation^5^. Del(20q) can occur as the sole cytogenetic abnormality or as the first cytogenetic abnormality in a more complex karyotype^6^.

The role of del(20q) as a primary recurrent cytogenetic abnormality in myeloid malignancies led to the hypothesis that one or more “myeloid” tumour suppressor genes (TSG) exist on chromosome 20q^7^. Indeed, mapping analyses of patient samples detected a frequently deleted region on chromosome 20q, named the “Common Deleted Region” (CDR). The CDR approximates 2.6 Mb in MPN samples and 2.7 Mb in MDS and AML samples, with an overlapping CDR region of 1.7 Mb^2^. Aziz and colleagues found that loss of L3MBTL1 and SGK2, two imprinted genes located in the CDR, resulted in hyperplasia of early erythroid progenitors but did not exclude a contribution from other genes located in the CDR to the del(20q) phenotype^8^.

Protein Tyrosine Phosphatase Receptor T (PTPRT) is a recognized TSG residing in the CDR. It is a member of the type IIb protein tyrosine phosphatase family. The extracellular segment of PTPRT contains a meprin-A5 antigen-PTP (MAM) domain, an immunoglobulin-like (Ig-like) domain, and four fibronectin type III (FNIII) repeats. The MAM, Ig domains and FNIII repeats are involved in cell adhesion^9–12^. The intracellular segment of PTPRT contains its catalytic region^13^, with the juxtamembrane domain followed by two phosphatase domains. The phosphatase D1 is responsible for the phosphatase activity, while the “pseudophosphatase” D2 is thought to modulate D1^14–17^. Recent studies demonstrated PTPRT’s role as a TSG in a wide range of human malignancies, including colorectal, lung and gastric cancers^13,14,18,19^. PTPRT is the most highly mutated tyrosine phosphatase in human colon carcinomas. Wang and colleagues^19^ examined 175 colorectal cancer samples for somatic mutations, and discovered that PTPRT mutations were the most common amongst 87 tyrosine phosphatase genes analysed. Moreover, PTPRT overexpression in these colorectal cancer cells inhibited their growth. PTPRT knockout mice have also been found to be highly susceptible to azoxymethane induced colon cancer^20^. Analyses of PTPRT mutations in lung, gastric and breast cancers^19,21^ suggested they were loss-of-function mutations resulting in diminished phosphatase activity. PTPRT mutations were shown to be common in head and neck squamous cell carcinomas by Lui and colleagues^22^. PTPRT mutation was also found in AML. Ley and colleagues used massively parallel sequencing technology to sequence a patient with AML. Eight heterozygous, non-synonymous somatic single nucleotide variants were detected, including PTPRT^23^.

We have reported on our analysis of patients with del(20q) MDS and AML using multicolour banding^5^. In addition to the CDR, these samples were demonstrated to have a “Common Retained Region” (CRR) at 20q11.2^24^. Further definition of the CRR using Fluorescence *In Situ* Hybridisation and array Comparative Genomic Hybridisation discovered tandem sequence amplification in five cases^5^. A 250 kb region represented the region of shortest DNA sequence with greatest amplification, up to 9 copies in one patient^5^. The CRR contained four complete genes of which the most likely candidate oncogene was the Src family protein tyrosine kinase Hemopoietic cell kinase (HCK). HCK has been implicated in AML, chronic myeloid leukemia (CML) and acute lymphoblastic leukemia^25^. HCK was found to be overactivated in Philadelphia positive murine myeloid cell line^26,27^ and there is evidence that HCK is required for BCR-ABL1 induced oncogenesis^28^. Pene-Dumitrescu and colleagues showed that HCK overexpression was sufficient to induce imatinib resistance in CML cells, apparently by direct phosphorylation of BCR-ABL1 by HCK^29^. HCK could also transmit antiapoptotic signals in CML, via BCR-ABL1 and STAT5. These data supported a role for HCK in BCR-ABL1-induced proliferation and survival^30^. Increased HCK expression and activation was also found in accelerated phase and blastic phase CML^31,32^. Furthermore, HCK has been shown to contribute to AML proliferation and survival. Dos Santos and colleagues reported that AML samples often co-expressed multiple activated Src family kinases, with most expressing both LYN and HCK. They also demonstrated that siRNA knockdown of HCK inhibited AML progenitor survival and proliferation. Lastly, HCK has also been shown to be highly differentially expressed in human primary AML stem cells compared to normal hemopoietic stem cells^33^, linking its presence in AML progenitors to recurrence of the disease.

Many oncoproteins have been identified to be the endogenous substrates of PTPRT and HCK. STAT3 (signal transducer and activator of transcription 3) is an oncoprotein involved in many hematological malignancies. HCK and PTPRT demonstrate opposing effect on STAT3 activity: PTPRT has been characterised as an important inhibitor of STAT3^34^ by dephosphorylation, while HCK phosphorylated and activated STAT3^35^.

Therefore, the abnormal chromosome 20 in del(20q) contained two regions of interest – the CDR and the CRR, that harbored the candidate TSG PTPRT, and the candidate oncogene HCK respectively. We hypothesized that PTPRT loss and HCK amplification cooperated to initiate myeloid malignancies, through the STAT3 pathway.

## Results

### HCK over-expression increases proliferative potential of HSCs

**Figure 1:**
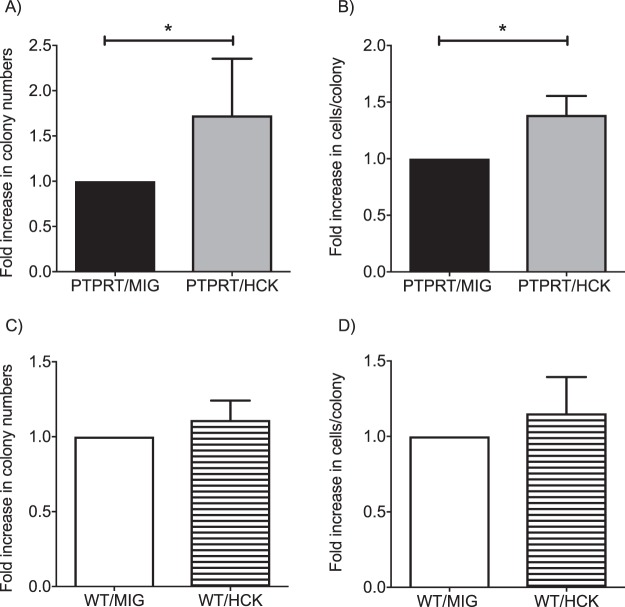
Hyperproliferation with over-expression of HCK in PTPRT−/− HSPCs. Over-expression of HCK in PRPRT−/− HSPCs (PTPRT/HCK) results in increased numbers of colonies (**A**) and colony size (**B**) compared with expression of control vector MIGR1 alone (PTPRT/MIG). Over-expression of HCK in wild type HSPCs (WT/HCK) does not augment colony numbers (**C**) or colony size (**D**) compared with expression of control vector alone (WT/MIG HSC). Data are shown as mean ± SD, n = 3–5. Statistical analysis: Student’s unpaired *t* test; *P < 0.05.

PTPRT−/− and wild type (WT) LKS+ haematopoietic stem and progenitor cells (LKS + HSPC) were retrovirally transduced with either HCK or control vector MIGR1 and cultured in methylcellulose media. Colony numbers and size (numbers of cells per colony) were enumerated. Over-expression of HCK only in PRTPRT−/− HSPCs demonstrated significantly higher colony numbers (Fig. 1A) and colony size (Fig. 1B). No proliferative advantage of HCK overexpression was noted in WT HSPCs (Fig. 1C,D). Cytospin analysis demonstrated normal myeloid differentiation with HCK overexpression in both PTPRT−/− and WT HSCs (data not shown).

### HCK expression increases STAT3 phosphorylation

**Figure 2:**
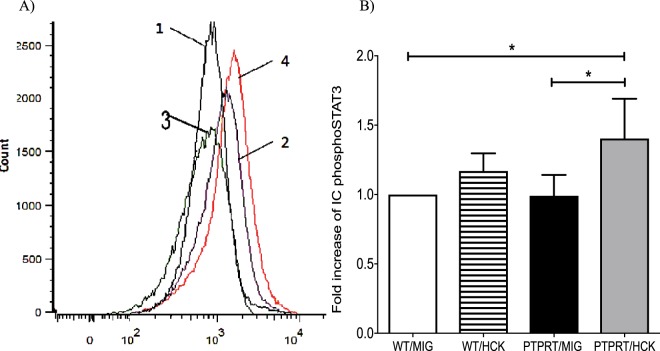
Increased Intracellular STAT3 phosphorylation with over-expression of HCK in PTPRT−/− HSPCs. Representative flow cytometry profile (**A**) and quantitative analysis (**B**) of Intracellular phosphoSTAT3 levels by mean APC intensity reveals maximal phosphoSTAT3 with HCK over-expression in PTPRT−/− HSPCs (PTPRT/HCK, line 4 in 2A) compared with overexpression of MIGR1 control vector in HSPCs of either genotype. Data are shown as mean ± SD, n = 5. Statistical analysis: one-way ANOVA with multiple comparisons; *P < 0.05.

We predicted that over-expression of HCK would lead to hyperphosphorylation of the second messenger STAT3 and this effect would be maximal in PTPRT−/− HSPCs as phosphoSTAT3 is a physiological substrate of PTPRT. The intracellular phosphoSTAT3 was quantified by flow cytometry. Figure 2A is representative of the effects of HCK over-expression on phosphoSTAT3 in either PTPRT−/− or WT HSPCs and quantification is shown in Fig. 2B. As expected, PTPRT/HCK HSPCs contained the highest level of intracellular phosphoSTAT3 with statistically significant augmentation compared to expression of control MIGR1 vector in PTPRT−/− and WT HSPCs. Over-expression of HCK in WT HSPCs did not lead to a significant increase in phosphoSTAT3 compared to MIGR1 vector alone confirming a synergistic effect of HCK on STAT3 hyperphosphorylation with absence of phosphatase activity with PTPRT deletion.

### Over-expression of HCK in PTPRT−/− HSCs results in splenomegaly and polycythemia

**Figure 3:**
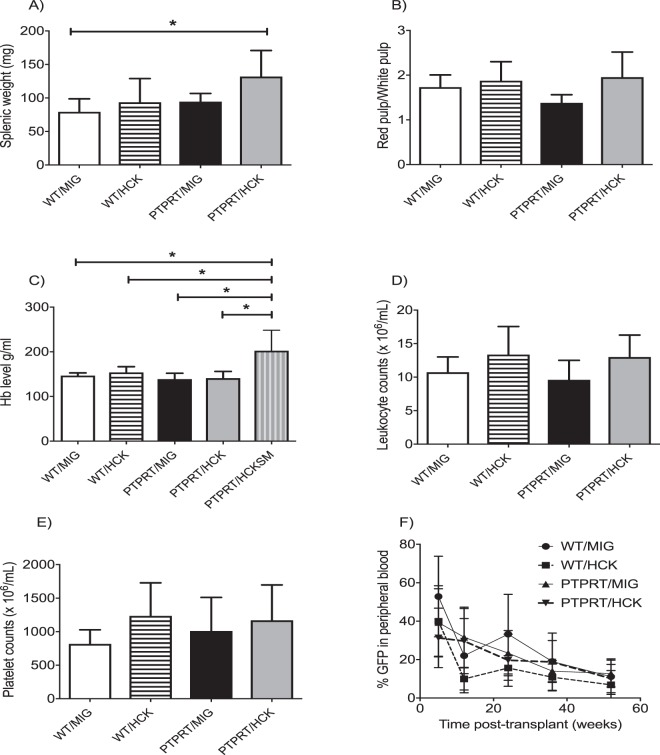
Development of splenomegaly and polycythemia in recipient mice transplanted with PTPRT−/− HSPCs with over-expression of HCK. (**A**) Significant increase in spleen weight in five out of 9 recipients reconstituted with PTPRT−/− HSPCs overexpressing HCK compared with WT HSPCs transduced with MIGR1 control vector. (**B**) Quantification of splenic red pulp versus white pulp by Osteomeasure after hematoxylin/eosin staining reveals no statistically significant red pulp expansion in recipients of PTPRT−/− HSPCs over-expressing HCK. (**C**) High hemoglobin noted in the cohort of PTPRT−/− HSPCs overexpressing HCK recipients with splenomegaly (SM) at 12 months compared to those with no splenomegaly (NS) and with other recipients. No significant difference in the peripheral blood leukocyte count (**D**) or platelet count (**E**) in any of the transplantation cohorts at 12 months. (**F**) No difference in GFP expression in cells of myeloid lineage in peripheral blood with over-expression of HCK in PTPRT−/− HSPCs at 12 months. Data are shown as mean ± SD, n = 4–9. Statistical analysis: one-way ANOVA with multiple comparisons except for (**F**): two-way ANOVA with multiple comparisons; *P < 0.05.

The oncogenic potential of HCK was explored *in vivo* by reconstituting sub-lethally irradiated CD45.1+ murine recipients with LKS+ HSPC from PTPRT−/− and WT mice retrovirally transduced with either HCK or MIGR1 control virus. All recipients were monitored up for 12 months and then culled to assess for features of abnormal hematopoiesis or leukemia. While PTPRT/HCK HSPC murine recipients did not develop overt myeloid leukemia, five out of nine recipients developed splenomegaly with the remaining four recipients demonstrating normal splenic weights (Fig. 3A). No morphological abnormalities were found with the other hematopoietic organs, such as livers and lymph nodes. Composition of red pulp and white pulp in the enlarged spleens was assessed by morphology with haematoxylin-eosin staining and quantified by Osteomeasure. There was no consistent difference noted between mice transplanted with either WT and PTPRT−/− HSPCs transduced with either MIGR1 control vector or HCK (Fig. 3B).

Full blood counts revealed that the five PTPRT−/− HCK-transduced HSPC murine recipients with splenomegaly were polycythemic (Fig. 3C), while the recipients without splenomegaly retained normal hemoglobin. However, both the leukocyte and the platelet counts did not reveal significant changes between mice transplanted with either WT and PTPRT−/− HSPCs transduced with either MIGR1 control vector or HCK (Fig. 3D,E).

Chimerism post transplantation was assessed by quantification of GFP reporter in peripheral blood leukocytes. The results showed a gradual decline over 12 months of monitoring without differences in mice transplanted with either WT and PTPRT−/− HSPCs transduced with either MIGR1 control vector or HCK (Fig. 3F). Apart from % GFP in the peripheral blood leukocytes, % GFP in bone marrow nucleated erythrocytes (at the 12 month analysis) were also analysed both in the primary as well as the secondary recipients (Supp Tables 1 and 2). % GFP in neither the peripheral blood leukocytes or bone marrow nucleated erythrocytes correlated with splenic weights.

### Aberrant bone marrow erythropoiesis in mice with splenomegaly reconstituted with PTPRT−/− HSCs transduced with HCK

**Figure 4:**
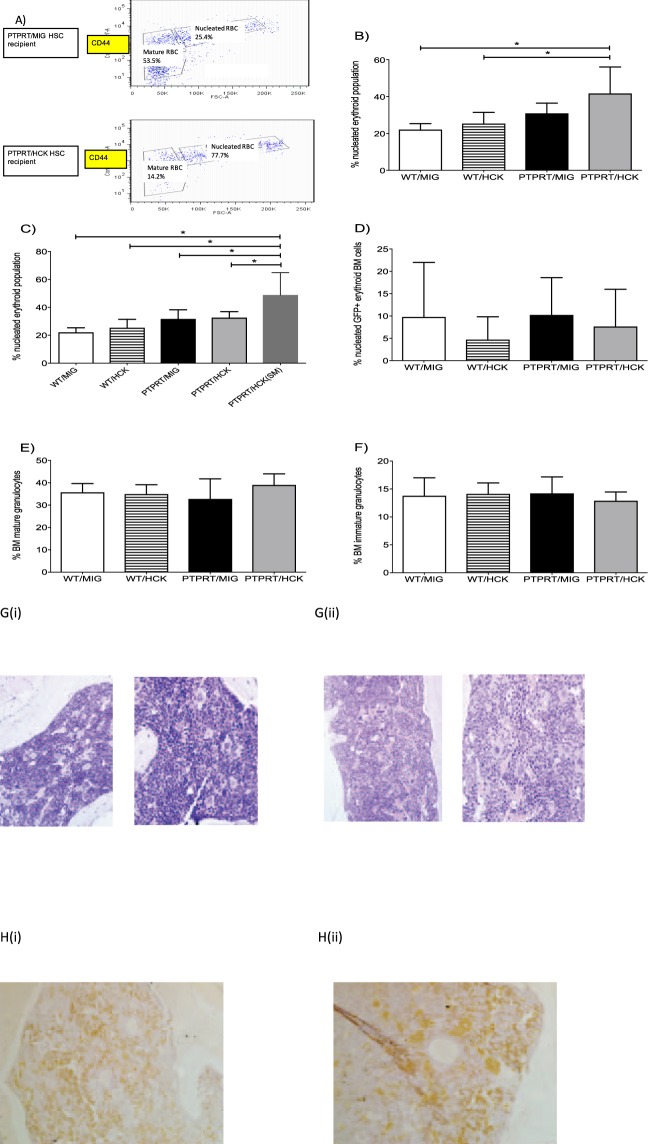
PTPRT/HCK HSPC recipients with splenomegaly have aberrant bone marrow erythropoiesis. (**A**) Representative flow cytometry profiles of resolution of bone marrow erythroid cells reveal relative abundance of nucleated red cells in PTPRT−/− HSPCs over-expressing HCK compared to MIGR1 control vector. (**B**) Bone marrow of mice reconstituted with PTPRT−/− HSPCs over-expressing HCK reveal a significant accumulation of nucleated red cells compared with WT HSPCs transduced with either HCK or MIGR1 control vector as assessed by flow cytometry. (**C**) PTPRT−/− HCK over-expressing recipients with splenomegaly (SM) reveal greater accumulation of nucleated red cells in bone marrow relative to those without splenomegaly as assessed by flow cytometry. (**D**) No difference in proportions of GFP-expressing nucleated red cells in recipients of either WT or PTPRT−/− HSPCs transduced with either HCK or MIGR1 control vector. No differences in bone marrow mature granulocytes (**E**) or immature granulocytes (**F**) between recipient mice transplanted with either WT or PTPRT−/− HSCs transduced with HCK or MIGR1 control vector. (**G**) Representative light microscopy images (magnification ×200 and ×400) after hematoxylin and eosin stain of femur sections from recipients transplanted with PTPRT−/− HSPCs overexpressing HCK (ii) and WT HSPCs overexpressing MIGR1 control vector (**i**) reveal normal tri-lineage hemopoiesis. (**H**) Immunohistochemistry to detect Ter119 of femur sections (left panel control, right panel PTPRTKO) do not reveal an accumulation of erythroid cells. Data are shown as mean ± SD, n = 4–9. Statistical analysis: one-way ANOVA with multiple comparisons; *P < 0.05.

The bone marrow erythroid population was resolved as either immature nucleated erythroid cells or mature erythrocytes based on expression of Ter119 and CD44 as described by Liu and colleagues(2). Figure 4A is a representative flow cytometry profile demarcating the nucleated erythroid cells and the mature RBC populations and demonstrates a higher proportion of nucleated erythroid cells in the bone marrow of recipients transplanted with PTPRT−/− HSPCs expressing HCK as compared with MIGR1 control virus. Over-expression of HCK in PTPRT−/− HSPCs was associated with greater accumulation of nucleated erythroid cells as compared with over-expression of HCK in WT HSPCs (Fig. 4B). Within the cohort of mice reconstituted with PTPRT−/− HSPCs, the shift towards immature erythroid cells was almost entirely in the mice with splenomegaly (Fig. 4C). Surprisingly, the proportion of GFP-positive nucleated erythroid cells was not higher in the PTPRT−/− HCK-transduced HSPC recipients (Fig. 4D) implying that the proportional increase in the early erythroid progenitors was probably due to an aberration in erythroid maturation or that there was a cell extrinsic effect of PTPRT−/− HCK on erythropoiesis.

Bone marrow myeloid maturation was resolved using Gr1 and CD11b expression into immature (Gr1 dim) and mature (Gr1 bright) granulocyte maturation. There was no difference between PTPRT−/− HCK-transduced HSPC recipients and other groups (Fig. 4E,F).

Morphology assessment of the bone marrow trephine paraffin sections after haematoxylin-eosin stain and TER119 immunohistochemistry showed that, despite the aberrant erythroid maturation in PTPRT/HCK HSPC recipients, there was no alteration in the overall bone marrow cellularity and normal trilineage haematopoiesis observed without evidence of leukemia, again indicating that the aberration is with maturation, not with the quantity (Fig. 4G,H).

### No perturbation in the stem cell or the committed progenitor populations in mice with reconstituted with PTPRT−/− HSPCs transduced with HCK

**Figure 5:**
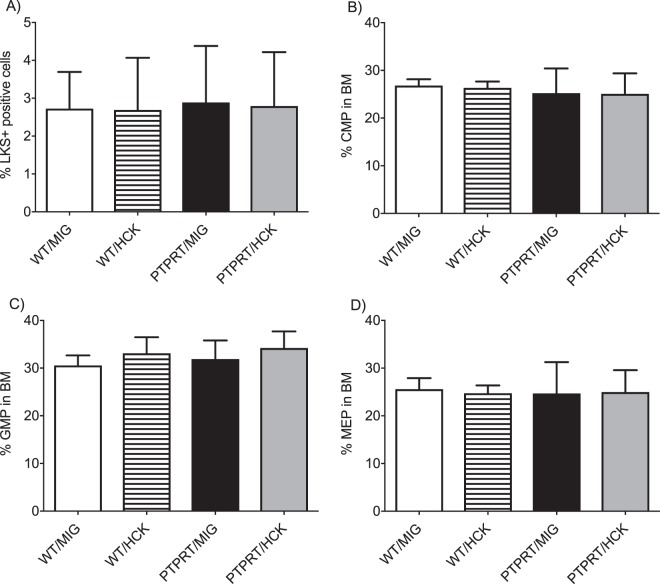
PTPRT/HCK HSPC recipients had normal LKS+ HSPC and LKS- progenitor populations. (**A**) % LKS+ HSPC of PTPRT/HCK HSPC recipients was similar to the other recipients. (**B**) % GMP of PTPRT/HCK HSPC recipients was similar to the other recipients. (**C**) % CMP of PTPRT/HCK HSPC recipients was similar to the other recipients. (**D**) % MEP of PTPRT/HCK HSPC recipients was similar to the other recipients. Data are shown as mean ± SD, n = 4–7. Statistical analysis: one-way ANOVA with multiple comparisons.

Mice were analysed at 12 months. Lineage negative cells were resolved as LKS+ and LKS- populations. There were no differences in the proportion of LKS+ cells between the PTPRT−/− HSPCs transduced with HCK and other recipients (Fig. 5A). LKS- progenitors were further categorised into Common Myeloid Progenitors (CMP), Granulocyte-Macrophage Progenitors (GMP) and Megakaryocyte-Erythroid Progenitors (MEP) using CD34 and CD16/32 expression and there were no differences in the progenitor populations between the various reconstitution cohorts (Fig. 5B,C,D).

### PTPRT/HCK HSC secondary recipients did not develop a leukemic phenotype

Since the primary murine recipients of PTPRT−/− HCK-transduced HSPCs demonstrated aberrant bone marrow erythropoiesis, splenomegaly and polycythemia without overt leukemia, BM HSPCs from the four cohorts were used to reconstitute lethally-irradiated secondary recipients. Two primary recipients of PTPRT−/− HSPCs transduced with either HCK or MIGR1 control at 7 months of age were used to harvest whole bone marrow cells for transplantation. The secondary recipients (three for each cohort) were monitored and then culled after 8 months. The analyses were identical to those of the primary recipients.

**Figure 6:**
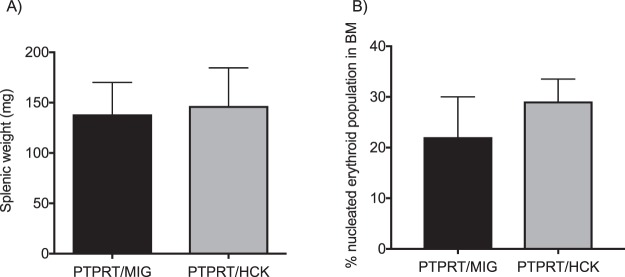
Secondary transplants of PTPRT/HCK HSPC recipients did not develop acute leukemia. (**A**) There was no difference in the splenic weights between the secondary PTPRT/HCK recipients and the control secondary PTPRT/MIG recipients. (**B**) There was no difference in the percentage of immature nucleated erythroid cells in the bone marrow between the secondary PTPRT/HCK recipients and the control secondary PTPRT/MIG recipients. Data are shown as mean ± SD, n = 3. Statistical analysis: one-way ANOVA with multiple comparisons.

Similar to the primary recipients, the examination of blood film, full blood counts, bone marrows and spleens did not demonstrate evidence of acute leukemia in the secondary recipients (data not shown). One out of three recipients of PTPRT−/− HSCs transduced with either HCK or MIGR1 control developed splenomegaly (Fig. 6A). Although there was a trend toward an expansion in proportion of immature erythroid cells in the PTPRT/HCK mice it did not reach statistical significance, possibly due to the small sample size (Fig. 6B).

## Discussion

Amplification of HCK and deletion of PTPRT are recurrent abnormalities in AML with 20q deletion. One possible mechanism of cooperation in oncogenic signalling is the hyperphosphorylation of STAT3 by HCK particularly in the absence of PTPRT, which serves as an endogenous brake by its capacity to dephosphorylate phospho-STAT3. We utilized retroviral transduction of HCK in PTPRT−/− HSPCs to provide *in vitro* evidence of signalling cooperation to result in proliferative advantage. In addition, we characterized the capacity of HCK amplification to lead to leukemia or MPN by reconstitution of mice with HSPCs overexpressing HCK.

There have been other published data that support our findings regarding a role for HCK and PTPRT in oncogenesis. HCK has been implicated in myeloid malignancies. In chronic myeloid leukemia, BCR-ABL1 activates several pro-survival substrates, including HCK, supporting a role for HCK in BCR-ABL1-induced proliferation and survival. Evidence suggests that BCR-ABL1 induced oncogenesis might require HCK, and HCK overexpression has been shown to be sufficient to induce imatinib resistance in CML. Increased expression and activation of HCK was also reported in accelerated phase or blastic phase CML. HCK has also been shown to contribute to AML proliferation and survival, as siRNA knockdown of HCK inhibited AML progenitor survival and proliferation. HCK has also been found to be highly differentially expressed in human primary leukemic stem cells compared with normal hematopoietic stem cells. The obvious importance of this finding is the contribution of AML progenitors to recurrence of the disease. Similarly, PTPRT mutation was also detected in AML using massively parallel sequencing technology to sequence the genomic DNA of both tumor and normal skin cells from a patient with AML. Many oncoproteins have been shown to be endogenous substrates of PTPRT, including STAT3. PTPRT has been characterised as an important direct inhibitor of STAT3, and aberrant STAT3 activation has been detected in a variety of hematopoietic malignancies such as AML.

In this study, overexpression of HCK was associated with increased STAT3 phosphorylation in HSPCs containing deletion of PTPRT. It was interesting to note that deletion of PTPRT alone was not adequate to cause hyperphosphorylation of STAT3 by endogenous murine HCK. These data correlated with alterations in colony numbers and size with significant increase noted only with over-expression of HCK in PTPRT−/− HSPCs and without proliferative advantage with over-expression of HCK in WT HSPCs that contain functional PTPRT. This raises the possibility that a double hit of HCK amplification and PTPRT loss is essential for providing proliferative advantage.

Our evidence demonstrates that PTPRT−/− HSPCs over-expressing HCK were hyperproliferative when cultured in methylcellulose media compared with colony numbers derived from both PTPRT−/− HSC with MIGR1 vector and WT HSCs over-expressing HCK. This correlated with higher intracellular phosphoSTAT3 in PTPRT−/− HSCs with HCK overexpression and is noteworthy because it represented hyperactivation of a well-recognised leukemogenic pathway. Therefore, an aberrantly activated STAT3 as a result of HCK amplification and PTPRT loss could be postulated as the driver mechanism for the myeloproliferative/leukemic phenotype noted in some del(20q) myeloid malignancies.

Our *in vivo* reconstitution experiment however did not recreate the phenotype of leukemia as seen in those patients with del(20q) who were initially studied to identify evidence of HCK amplification. Leukemia was not noted even in mice reconstituted with PTPRT−/− HSPCs overexpressing HCK even after 12 month post primary transplantation and after secondary transplantation. However, five out of nine mice did exhibit an interesting phenotype of abnormally high bone marrow immature nucleated erythroid cells, splenomegaly and polycythemia. The three characteristics appeared to be strongly correlated and supports the hypothesis that HCK amplification on the background of PTPRT depletion had directly resulted in aberrant bone marrow erythroid development, which subsequently led to the establishment of polycythemia and splenomegaly. These findings resembled signs associated with MPN, specifically polycythemia vera (PV), a myeloid malignancy that is most frequently associated with del(20q). Interestingly, two out of the five del(20q) patients that were examined to map out the CRR by MacKinnon and colleagues had erythroleukemia(5), however, this was not observed in the mice.

There are some caveats to our findings. Firstly, in the affected PTPRT/HCK HSPC recipients, the myeloproliferative phenotype was incomplete – the bone marrow cellularity and hematopoiesis were normal, and the bone marrow erythroid population was not increased. Secondly, the phenotype was not fully penetrant, with five out of nine PTPRT/HCK HSPC recipients developing the aberrant features. Finally, the decreasing percentage of peripheral GFP positive cells suggested that PTPRT−/− HCK-overexpression did not confer a survival advantage to HSPCs. This posed an interesting question as to the mechanisms behind these myeloproliferative changes. Although outside the scope of our study, several mechanisms have been proposed: firstly, autocrine or paracrine cytokine production by the PTPRT/HCK HSC could have contributed to these changes. These cytokines could have an effect on the neighbouring non-GFP positive cells by promoting an immature erythroid development. Secondly, the bone marrow microenvironment played an important role in the maturation of hematopoietic cells. Whether some of the PTPRT/HCK HSC transformed into support cells in the microenvironment or influenced the non-hematopoietic bone marrow microenvironment cells to alter their support potential and thereby had an influence on the differentiation of the erythroid population would also be an informative question to answer, but is beyond the scope of this study. Finally, hematological malignancies such as acute myeloid leukemia with 20q loss usually have other relevant copy number abnormalities, hence other mutations are likely needed in addition to our model, explaining the partial penetrance in the mice.

There is considerable heterogeneity in human disease with del(20q) as a sole abnormality being recognised as a favorable cytogenetic prognostic marker in MDS. Transformation to leukemia in some del(20q) patients implies the presence of additional events and one of these could be variability in HCK amplification in terms of copy numbers. All mice reconstituted with PTPRT−/− HCK-over-expressing HSPCs revealed hyperphosphorylation of STAT3 but the precise copy numbers of HCK were not ascertained and it is possible that there is a gene dosage effect that is necessary to establish a MPN/leukemia phenotype. Furthermore, we cannot exclude a contribution from other candidate TSG located in the CDR, such as L3MBTL1 and SGK2, to the del(20q) phenotype, as described in other contiguous gene deletion syndromes like the 5q- syndrome.

There is variability of phenotypes in JAK2 mutation-containing myeloproliferative neoplasms. PV is the myeloid malignancy that is most frequently associated with del(20q), and is characterized by erythrocytosis. Almost all patients with polycythemia vera have a gain-of-function mutation in the Janus kinase 2 gene (JAK2) with the resultant constitutively activated JAK/STAT signaling pathway causing the MPN phenotype. Allele burden of the JAK2 mutation also has an influence on disease phenotypes seen in polycythemia vera versus essential thrombocythemia, another related MPN in which about 50% of patients would harbor the JAK2 mutation.

Possible reasons why the combination of PTPRT depletion and HCK overexpression has an effect only on an erythroid lineage include: Firstly, the level of hyperphosphorylation, and consequent variations in aberrant activation of the STAT3 pathway, might have a crucial role in the phenotype of the murine recipients. Therefore, a higher level of STAT3 hyperphosphorylation and activation might lead to phenotype penetrance on other hematopoietic linages. The second reason could be that the combination of genetic changes and effect of signalling on erythropoiesis via the EPO pathway, may have skewed towards an erythroid phenotype, though this was unable to be delineated in our work.

To summarize, the combination of HCK overexpression and PTPRT loss was associated with some myeloproliferative characteristics such as hyperproliferation and STAT3 upregulation *in vitro*, and aberrant bone marrow erythroid maturation, polycythemia and splenomegaly *in vivo*. We surmised that the dysregulation of these two genes in some del(20q) malignancies at least have a contribution in the development of a myeloproliferative neoplasm, such as PV.

## Methods

All animal experiments were approved and conducted in accordance with the guidelines of the St. Vincent’s Health Animal Ethics Committee.

### Harvesting and retroviral transduction of murine LKS+ hematopoietic stem cells

Ficoll-Paque was used to collect mononuclear cells from C57BL6 wild type (WT) and PTPRT-null (PTPRT) mice and LKS+ hematopoietic stem and progenitor cells (HSPC) were collected by FACS sorting. Retroviral transduction of either MIG control or HCK was performed using Retronectin spinofection. 500 μl of Retronectin (40 μg/ml) was added to four wells (WT/MIG, WT/HCK, PTPRT/MIG and PTPRT/HCK) on a 24-well suspension culture plate and incubated overnight at 4 °C. Excess Retronectin was removed, and the Retronectin-coated wells were blocked using 300 μl of PBS + 2% BSA per well. This was incubated at room temperature for 30 minutes. The blocking solution was removed, and the wells were washed twice with PBS. Then either MIG or HCK retroviral supernatant was added to the wells (multiplicity of infection of 10x). The suspension plate was incubated at 37 °C for one hour, then centrifuged 1100 g at room temperature for two hours. The retroviral supernatant from the suspension plate was removed and 2 × 10^4^ LKS+ HSPC were added to each well. The HSC-Retronectin suspension plate was centrifuged at 400 g for 1.5 hours and then incubated at 37 °C. Cells with either MIG or HCK transduced were sorted by FACS (GFP positive).

### Methylcellulose colony assay

WT/MIG HSC, WT/HCK HSC, PTPRT/MIG HSC, and PTPRT/HCK HSC were resuspended in PBS to 50 cells/μL (5 × 10^4^/ml). 2.5 ml of methylcellulose media was aliquoted into four tubes, one for each cell type, to which 25 μl of cells was added. The media contained recombinant murine SCF and IL-3, as well as recombinant human IL-6. The tubes were mixed and then 1 ml of mixture was plated per 35 mm petri dish. Duplicate plates and an extra 35 mm dish with sterile PBS (maintain moisture) were also placed in the 100 mm petri dish and incubated at 37 °C. The colonies (>50 cells) were counted at day 7 and 12. The total number of cells per dish were also enumerated by dissolving the methylcellulose media using PBS and counting the cells.

### Intracellular PhosphoSTAT3 antibody assay

WT/MIG HSC, WT/HCK HSC, PTPRT/MIG HSC, and PTPRT/HCK HSC were suspended in 200 μl of StemPro-34 media @ 37 °C. 1 ml pre-warmed 1% buffered formalin was added and incubated for 10 minutes at 37 °C. 1 ml of ice-cold PBS was added for wash twice. The cells were resuspended in 500 μl ice-cold 90% MeOH and incubated for 30 minutes on ice. The cells were washed in PBS + 0.5% BSA and then resuspended in 1 ml of PBS + 0.5% BSA for 30 minutes on ice. The tubes were centrifuged and resuspended in 40 μl of PBS + 0.5% BSA. 10 μl of STAT3-APC antibody was added to each tube and incubated for one hour on ice. The tubes were then washed twice with PBS + 0.5% BSA and then resuspended for flow cytometry.

### *In vivo* reconstitution assay

Two days post retroviral transduction, the GFP percentage of LKS+ HSPC was measured. 20,000 LKS+ HSPC and 200,000 LKS- myeloid progenitors were washed using PBS and tail-vein injected into each B6.SJL/PTPRCa murine recipient. These recipients were bled at one month, and then every 3 months until 12 months post transplantation for monitoring. All the murine recipients were culled and analysed at 12 months post transplantation. Secondary reconstitution assay was also performed. Two of each of PTPRT/HCK HSC and PTPRT/MIG HSC recipients were culled at approximately 7 months post transplant and 5 × 10^6^ bone marrow cells transplanted into new secondary recipients. The secondary recipients were monitored and then culled and analyzed at 8 months of age.

### Cytospin analysis

The cytospin was performed using a Shandon Cytospin 2 cytocentrifuge. Labeled slides were inserted into the cytocentrifuge chambers. Cell suspensions were then made from the methylcellulose colonies selected using 200 ul of FACS buffer (PBS + 2% FCS). The cell suspensions were added to the cytocentrifuge, and spun at 600 rpm for 10 minutes. The cells were then transferred to the slides in the chambers. The cytospin slides were removed from the cytocentrifuge and allowed to dry. They were stained with hemotoxylin and eosin and analyzed by light microscopy with regards to their morphology.

## Supplementary information

Dataset 1, 2
