# Supplementary material for: Hemopoietic Cell Kinase amplification with Protein Tyrosine Phosphatase Receptor T depletion leads to polycythemia, aberrant marrow erythoid maturation, and splenomegaly
Source: Sci Rep. 2019 May 7;9:7050. doi: 10.1038/s41598-019-43373-6 (PMC6505535; doi:10.1038/s41598-019-43373-6)
Supplement: Supplementary file 1 — Dataset 1, 2 [file 41598_2019_43373_MOESM1_ESM.docx]

**Hemopoietic Cell Kinase amplification with Protein Tyrosine Phosphatase Receptor T depletion leads to polycythemia, aberrant marrow erythoid maturation, and splenomegaly**

# Matthew Ku^1,4^, Ruth N. MacKinnon^2^, Meaghan Wall^2^, Nisha Narayan^1^, Carl Walkley^3,4^, Heung-Chin Cheng^5^, Lynda J Campbell^2^, Louise E Purton^3,4^* and Harshal Nandurkar^4,6^* (*Joint senior authors)

^1^Department of Haematology, St Vincent's Hospital, Fitzroy, Australia 3065

^2^Victorian Cancer Cytogenetics Services, St Vincent’s Hospital, Fitzroy, Australia 3065

^3^St Vincent’s Institute of Medical Research, Fitzroy, Australia 3065

^4^Department of Medicine, St Vincent’s Hospital, The University of Melbourne, Fitzroy, Australia 3065

^5^The University of Melbourne, Parkville, Australia 3010

^6^The Australian Centre for Blood Diseases, Monash University, Melbourne, Australia 3004

**Supplemental Tables**

Supplemental Table 1


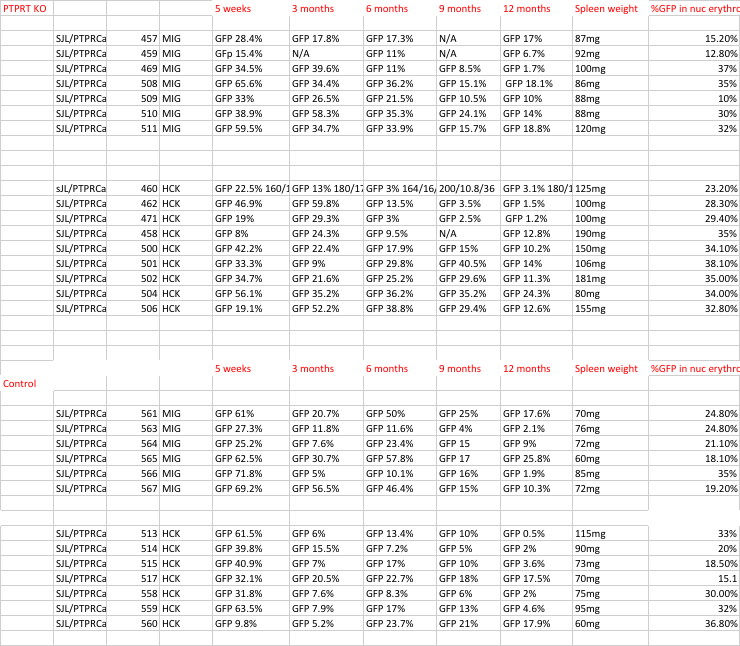


Supplemental Table 2

|  |  | 5 weeks | 3 months | 6 months | Spleen weight | %GFP in nuc erythrocytes |
| --- | --- | --- | --- | --- | --- | --- |
| MIG/PTPRT | 833 | GFP 50% | GFP 50.7% | GFP 49% | 120mg | 11.80% |
|  | 834 | GFP 37.3% | GFP 39% | GFP 37.5% | 170mg | 25.20% |
|  | 836 | GFP 48.5% | GFP 47.2% | GFP 40% | 70mg | 13% |
|  |  |  |  |  |  |  |
| HCK/PTPRT | 838 | GFP 13.6% | GFP 13.4% | GFP 14% | 130mg | 16% |
|  | 839 | GFP 15.8% | GFP 15.8% | GFP 25.5% | 160mg | 20% |
|  | 840 | GFP 12.8% | GFP 13.6% | GFP 16.7% | 110mg | 22.40% |

**Table Legends**

**Table 1. Chimerism table for the primary recipients**

% GFP in peripheral blood leukocytes at different timepoints of analysis post-transplantation, % GFP in bone marrow nucleated erythrocytes at the final 12 month analysis, and the splenic weights.

**Table 2. Chimerism table for the secondary recipients**

% GFP in peripheral blood leukocytes at different timepoints of analysis post-transplantation, % GFP in bone marrow nucleated erythrocytes at the final 12 month analysis, and the splenic weights.
